# Evidence-based practice in Behçet’s disease: identifying areas of unmet need for 2014

**DOI:** 10.1186/1750-1172-9-16

**Published:** 2014-01-30

**Authors:** Robert J Barry, Bharat Markandey, Rahul Malhotra, Henry Knott, Nikita Joji, Mohammed Mubin, Alastair K Denniston, Phillip I Murray

**Affiliations:** 1 Centre for Translational Inflammation Research, School of Immunity and Infection, College of Medical and Dental Sciences, Queen Elizabeth Hospital, University of Birmingham, B15 2TT Birmingham, UK; 2 Academic Unit of Ophthalmology, University of Birmingham, Birmingham and Midland Eye Centre, City Hospital, Birmingham, UK; 3 University of Birmingham Medical School, University of Birmingham, Birmingham, UK; 4 Department of Ophthalmology, Queen Elizabeth Hospital Birmingham, Edgbaston, Birmingham, UK; 5 Behçet’s Centre of Excellence, City Hospital, Sandwell & West Birmingham Hospitals NHS Trust, Birmingham, UK

**Keywords:** Behçet’s disease, Evidence based medicine, Therapy, Systematic review

## Abstract

**Background:**

Behçet’s Disease (BD) is characterized by a relapsing-remitting course, with symptoms of varying severity across almost all organ systems. There is a diverse array of therapeutic options with no universally accepted treatment regime, and it is thus important that clinical practice is evidence-based. We reviewed all currently available literature describing management of BD, and investigated whether evidence-based practice is possible for all disease manifestations, and assessed the range of therapeutic options tested.

**Methods:**

We conducted an internet search of all literature describing management of BD up to August 2013, including pharmacological and non-pharmacological interventions. We recorded treatment options investigated and disease manifestations reported as primary and secondary study outcomes. Quality of data was assessed according to the Scottish Intercollegiate Guideline Network (SIGN) hierarchy of evidence.

**Results:**

Whilst there is much literature describing treatment of ocular and mucocutaneous disease, there is little to guide management of rheumatoid, cardiovascular and neurological disease. This broadly reflects the prevalence of disease manifestations of BD, but not the severity. Biologic therapies are the most commonly investigated intervention. The proportion of SIGN-1 graded studies is declining, and there are no SIGN-1 graded studies investigating neurological or gastrointestinal manifestations of BD.

**Conclusions:**

This is the first study to investigate trends in published literature for management of BD over time. It identifies neurological, cardiovascular and gastro-intestinal disease as particular areas of unmet need and suggests that overall quality of evidence is declining. Future research should be designed to address these areas of insufficiency to facilitate evidence-based practice in BD.

## Background

To achieve optimum patient outcomes, clinicians must strive to practice evidence-based medicine wherever possible. Evidence-based practice has two requirements: the availability of published research [1], and the ability to critically appraise the information presented [2].

Decisions regarding treatment should thus be informed by the experience of others, as presented in peer-reviewed medical and scientific journals. Clinicians must however be aware of bias and inaccuracy, and be prepared to question such literature. Publication does not guarantee quality, and there is a hierarchy of evidence ranging from that derived through expert opinion and case report, to that supported by large-scale randomized controlled trials [3].

This is particularly important for the management of rare diseases, where there may not be a widely-accepted “gold-standard” treatment, and also in complex multi-system diseases, where there are likely to be a range of potential treatment options depending on the presenting features. Behçet’s Disease (BD) is both rare and complex: is a multi-system inflammatory disorder of unknown aetiology [4], with an estimated incidence of 0.64 per 100,000 people in the UK and 5.2 per 100,000 in the USA. Higher disease rates are observed in Mediterranean and Far Eastern countries along the historic “Silk Route”, with disease incidence in Turkey between 20 and 421 per 100,000 people [5].

Patients display a diverse spectrum of symptoms, both in terms of organ system affected and also the severity of disease in each of these systems [6]. As a result, disease management is variable with therapeutic options ranging from symptomatic relief through to systemic immunosuppression [7,8]. Treatment is usually instigated and monitored by a multi-disciplinary team, involving collaboration between dermatologists, ophthalmologists and rheumatologists, with input by cardiologists, genitourinary physicians and neurologists as required.

It follows that best practice in BD demands a large body of supporting literature; it is important that each member of the multi-disciplinary team has access to up-to-date evidence to guide their management, with supporting data for each potential disease manifestation, at varying degrees of severity.

We set out to investigate the scope and quality of the evidence-base available to clinicians involved in the management of BD, paying particular attention to how this literature has evolved over time. We were keen to determine if it is possible to practice evidence-based medicine for all potential disease manifestations and through review of observed trends to identify any areas of unmet need, to guide further research in this area. This was achieved through a comprehensive review of all available peer-reviewed literature, assessing the range of therapeutic options investigated, the disease manifestations for which these investigations were performed and the overall quality of the resulting research.

## Methods

A systematic online literature search was performed using the PubMed database, Medline, EMBASE and the Cochrane Central Register of Controlled Trials (CENTRAL) for all studies published before August 2013 combining the terms “therapy OR therapeutic OR treatment”, “behçet*” (exploded), and all publication types relating to clinical trials as listed in the PubMed database.

To be considered for further review, studies were assessed against strict inclusion and exclusion criteria; for inclusion, all documented cases of BD must have been diagnosed according to the International Study Group (ISG) guidelines (1990) [9], or for those studies recruiting patients prior to the publication of these guidelines, diagnosis of BD must have been deemed concordant with ISG criteria by all authors of this review. Furthermore, the study must have been directly concerned with a therapeutic intervention. Both pharmacological and non-pharmacological interventions were included in the review.

Publications were excluded if the intervention group comprised fewer than 20 patients with ISG-confirmed BD, or if the study did not directly assess a therapeutic option. Duplicates, narrative reviews and editorials were excluded from further analysis. Due to the native language of the reviewers, we were unable to assess studies without an English language translation. Since our aim was to assess the range of data currently available, previous meta-analyses and systematic reviews were also excluded from the quantitative analysis.

Data extracted comprised date of publication, number of patients with ISG-confirmed BD, disease manifestations investigated and whether this was as a primary or secondary study outcome, and therapeutic intervention(s) assessed. Quality was assessed in accordance with the Scottish Intercollegiate Grading Network (SIGN) hierarchy of evidence grading for published literature [3], using a simplified categorization as indicated in Table 1. Data was entered into a Microsoft Excel spreadsheet for coding and further analysis.

**Table 1:** Scottish Intercollegiate Guideline Network (SIGN) grading of evidence, with equivalent simplified categorization used for present analysis

**Level of evidence**	**Description**	**Categorisation in this analysis**
1++	High quality meta-analyses, systematic reviews of RCTs, or RCTs with a very low risk of bias	1
1+	Well conducted meta-analyses, systematic reviews of RCTs, or RCTs with a low risk of bias	
1-	Meta-analyses, systematic reviews or RCTs, or RCTs with a high risk of bias	
2++	High quality systematic reviews of case-control or cohort studies or High quality case-control or cohort studies with a very low risk of confounding, bias, or chance and a high probability that the relationship is causal	2
2+	Well conducted case-control or cohort studies with a low risk of confounding, bias, or chance and a moderate probability that the relationship is causal	
2-	Case-control or cohort studies with a high risk of confounding, bias, or chance and a significant risk that the relationship is not causal	
3	Non-analytic studies, e.g. case reports, case series	3
4	Expert opinion	4

## Results

The initial search identified 255 papers, of which 60 met the above criteria for further review (Table 2). Most studies were excluded due to small sample size.

**Table 2:** List of papers included in analysis, arranged in date order (most recent first) with PubMed PMID reference numbers

	**Title**	**Published**	**PubMed PMID**
[10]	Health- and vision-related quality of life in patients receiving infliximab therapy for Behcet uveitis	2013	23314623
[11]	Secukinumab in the treatment of noninfectious uveitis: results of three randomized, controlled clinical trials		23290985
[12]	Clinical background comparison of patients with and without ocular inflammatory attacks after initiation of infliximab therapy	2012	23053631
[13]	Multicenter study of infliximab for refractory uveoretinitis in Behçet disease		22652845
[14]	A single infliximab infusion vs corticosteroids for acute panuveitis attacks in Behçet’s disease: a comparative 4-week study	2011	21097877
[15]	One year study of efficacy and safety of infliximab in the treatment of patients with ocular and neurological Behçet’s disease refractory to standard immunosuppressive drugs		19859715
[16]	Azathioprine in severe uveitis of Behçet’s disease	2010	20665749
[17]	Comparison of infliximab versus ciclosporin during the initial 6-month treatment period in Behçet disease		20545993
[18]	Effects of atorvastatin and lisinopril on endothelial dysfunction in patients with Behçet’s disease		20704623
[19]	Pimecrolimus versus placebo in genital aphthous ulcers of Behcet’s disease: a randomized double-blind controlled trial		20704622
[20]	Rituximab in intractable ocular lesions of Behcet’s disease; randomized single-blind control study (pilot study)		19692382
[21]	Colchicine versus placebo in Behçet’s disease: randomized, double-blind, controlled crossover trial	2009	19076988
[22]	Effectiveness and safety of endovascular aneurysm treatment in patients with vasculo-Behçet disease		19717547
[23]	Low-dose natural human interferon-alpha lozenges in the treatment of Behçet’s syndrome		19842735
[24]	Randomized trial of pimecrolimus cream plus colchicine tablets versus colchicine tablets in the treatment of genital ulcers in Behçet’s disease		19434815
[25]	Relationship between periodontal parameters and Behçet’s disease and evaluation of different treatments for oral recurrent aphthous stomatitis		19320802
[26]	The close association between dental and periodontal treatments and oral ulcer course in behcet’s disease: a prospective clinical study		19060462
[27]	Treatment with levamisole and colchicine can result in a significant reduction of IL-6, IL-8 or TNF-α level in patients with mucocutaneous type of Behcet’s disease		19597921
[28]	Behçet’s disease: comparing 3 decades of treatment response at the National Eye Institute	2008	18929351
[29]	Infliximab effects compared to conventional therapy in the management of retinal vasculitis in Behçet disease		18711463
[30]	Interrelated modulation of endothelial function in Behcet’s disease by clinical activity and corticosteroid treatment	2007	17845731
[31]	A double-blind trial of depot corticosteroids in Behçet’s syndrome	2006	17067433
[32]	Lactobacilli lozenges in the management of oral ulcers of Behçet’s syndrome		16923135
[33]	Oral zinc sulfate in the treatment of Behcet’s disease: a double blind cross-over study		16263779
[34]	Colchicine and benzathine penicillin in the treatment of Behçet disease: a case comparative study	2005	16409899
[35]	Short-term trial of etanercept in Behçet’s disease: a double blind, placebo controlled study		16321889
[36]	Vitrectomy for persistent panuveitis in Behçet’s disease		15630733
[37]	Abdominal aortic aneurysm in Behçet’s disease: new treatment options for an old and challenging problem	2004	15079762
[38]	Differential efficacy of human recombinant interferon-α2a on ocular and extraocular manifestations of behçet disease: results of an open 4-center trial		14996689
[39]	Infliximab for recurrent, sight-threatening disease in Adamantiades-Behcet disease		15055270
[40]	Clinical study on therapeutic effect of acupuncture on Behcet’s disease	2003	14719295
[41]	Efficacy of rebamipide as adjunctive therapy in the treatment of recurrent oral aphthous ulcers in patients with Behçet’s disease: a randomised, double-blind, placebo-controlled study		12928692
[42]	Evaluation of the effect of acetazolamide on cystoid macular oedema in patients with Behcet’s disease		12918754
[43]	Dapsone in Behçet’s disease: a double-blind, placebo-controlled, cross-over study	2002	12568631
[44]	A double-blind trial of colchicine in Behçet’s syndrome	2001	12081158
[45]	Mycophenolate mofetil is ineffective in the treatment of mucocutaneous Adamantiades-Behçet’s disease		11935432
[46]	Short-term chlorambucil for refractory uveitis in Behcet’s disease		11710724
[47]	Ciclosporin Microemulsion Preconcentrate Treatment of Patients With Behçet’s Disease	1999	11752821
[48]	Effects of interferon-alpha2a treatment on serum levels of tumor necrosis factor-alpha, tumor necrosis factor-alpha2 receptor, interleukin-2, interleukin-2 receptor, and E-selectin in Behçet’s disease		10482480
[49]	The Use of Sucralfate Suspension in the Treatment of Oral and Genital Ulceration of Behchet Disease		10328192
[50]	Thalidomide in the Treatment of the Mucocutaneous Lesions of the Behcet Syndrome	1998	10651074
[51]	Treatment of Adamantiades-Behçet disease with systemic interferon alfa		9722733
[52]	Effect of prophylactic benzathine penicillin on mucocutaneous symptoms of Behçet’s disease	1996	9499327
[53]	Interferon alfa-2a in the treatment of Behçet’s disease		8829493
[54]	A phase II study of FK506 (tacrolimus) on refractory uveitis associated with Behçet’s disease and allied conditions	1995	8594659
[55]	Inefficacy of azapropazone in the acute arthritis of Behçet’s syndrome: a randomized, double blind, placebo controlled study		7586783
[56]	Clinical experience with thalidomide in the management of severe oral and genital ulceration in conditions such as Behçet’s disease: use of neurophysiological studies to detect thalidomide neuropathy	1994	7864692
[57]	Systemic interferon alpha 2b treatment in Behçet’s syndrome		7932420
[58]	Visual prognosis in patients with Behçet’s disease receiving colchicine, systemic corticosteroid or cyclosporin		7970549
[59]	Low dose cyclosporin A versus pulsed cyclophosphamide in Behçet’s syndrome: a single masked trial	1992	1390495
[60]	Desensitization by autologous saliva and Behçet’s disease	1991	1913022
[61]	Effect of cyclosporine A on the hearing loss in Behçet’s disease		1771312
[62]	Inefficacy of Topical Alpha Interferon in the Treatment of Oral Ulcers of Behcet’s Syndrome: a Randomized Double Blind Trial		2058987
[63]	Long-term effects of cyclophosphamide and colchicine treatment in Behçet’s disease		2064258
[64]	Treatment of Behçet disease with indomethacin		1993568
[65]	A controlled trial of azathioprine in Behcet’s syndrome	1990	2404204
[66]	Topical alpha interferon in the treatment of oral ulcers in Behçet’s syndrome: a preliminary report		2189624
[67]	Double-masked trial of cyclosporin versus colchicine and long-term open study of cyclosporin in behcet’s disease	1989	2566048
[68]	Evaluation of conventional therapy versus cyclosporine A in Behçet’s syndrome	1988	3044474
[69]	Treatment with aciclovir does not affect orogenital ulcers in Behcet’s syndrome: a randomised double-blind trial		3381269

Full list of references included at end of main text [10-69].

Of the 60 studies included in this analysis, ocular and mucocutaneous manifestations of disease were the most frequently reported primary outcomes (27 and 26 studies respectively), with far fewer studies reporting on cardiovascular, neurological or rheumatological outcomes (Table 3). Two studies were primarily concerned with quality of life measures, and two with serological markers of disease with no symptomatic correlation. Eight studies (13.33%) reported on a range of disease manifestations as primary outcomes.

**Table 3:** Outcomes of studies included in review, stratified by primary and secondary measures

**Disease manifestation**	**Number of studies**	
	**Primary outcome**	**Secondary outcome**
Ocular	27	2
Mucocutaneous	26	5
Rheumatoid	8	8
Cardiovascular	4	5
Neurological	2	5
Other	4	6

These studies represent an overall sample size of 4302 patients with BD, of which 1555 were involved in studies reporting on ocular outcomes, 1420 mucocutaneous outcomes, 1144 cardiovascular outcomes, 449 rheumatological outcomes, 56 neurological, and 109 other outcomes.

The most commonly reported secondary outcome was rheumatological disease, being assessed in eight studies, with mucocutaneous disease, cardiovascular and neurological disease each being reported as secondary outcome in a further five studies.

A total of 37 distinct therapeutic interventions were assessed, which were categorized as shown in Table 4. Most studies investigated pharmacological agents, with only six studies investigating non-pharmacological interventions (ocular surgery, retinal laser, cardiovascular surgery, interventional radiology, dental hygiene and dental surgery).

**Table 4:** Interventions assessed by studies included in review

**Intervention**	**Number of studies**
Biologic	20
Corticosteroid	8
Antiproliferative	5
Alkylating agent	4
Calcineurin antagonist	12
Other immune modulator	12
NSAID	2
Anti-bacterial	4
Drug acting on cardiovascular system^a^	2
Other pharmacological intervention^b^	5
Non-pharmacological intervention^c^	6

^a^ACEI and statins.

^b^Autologous saliva, lactobacilli lozenges, rebamipide, sucralfate, zinc sulphate.

^c^Ocular surgery/laser, cardiovascular surgery/laser, dental surgery/hygiene.

**Figure 1:**
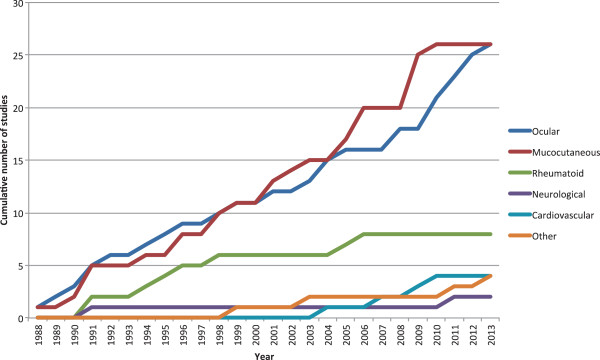
Disease manifestation reported as primary study outcome 1988-2013.

Between 1988 and 2013, there has been a steady increase in the number of publications reporting on ocular and mucocutaneous disease manifestations as primary outcome measures. During this period, there has been a slower increase in the number of studies reporting on rheumatological disease as primary outcome and little increase in the number of studies reporting on all other manifestations (Figure 1).

**Figure 2:**
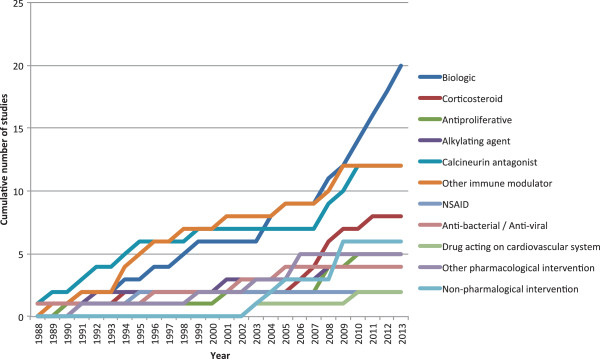
Therapeutic intervention assessed 1988-2013.

**Figure 3:**
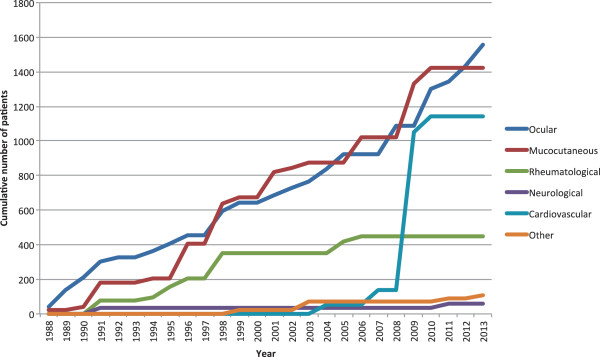
Use of biologic therapy 1988-2013, stratified by disease manifestation reported as primary study outcome.

Since 2007 there has been a significant increase in the number of studies investigating biologic therapies for manifestations of BD (Figure 2). The most significant increase has been in those studies reporting on ocular manifestations as primary outcome, for which the cumulative number of studies has increased by 140% since 2009 (Figure 3).

**Figure 4:**
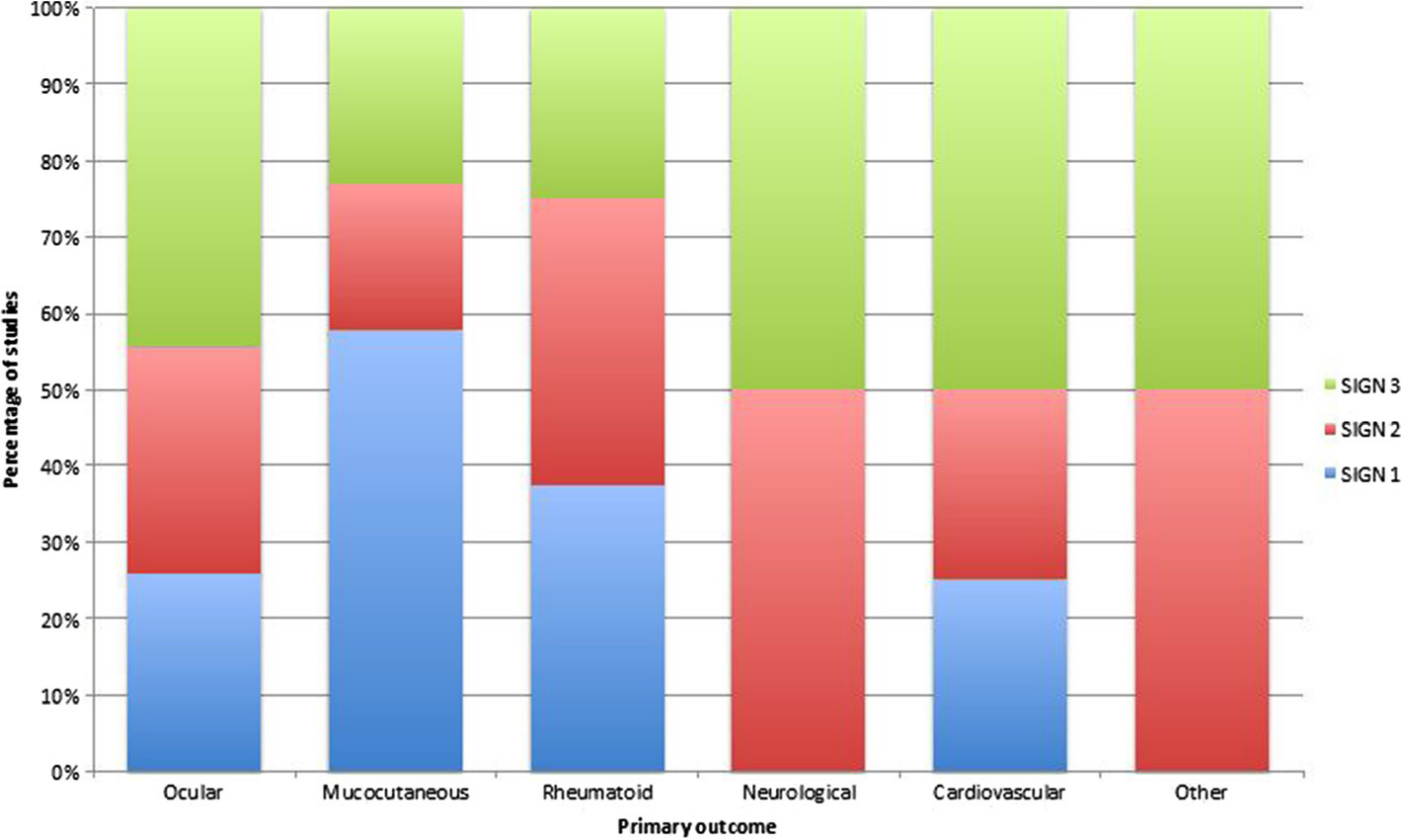
SIGN score by disease manifestation reported as primary study outcome 1988-2013.

According to the SIGN grading for hierarchy of evidence [3], the highest quality research has been conducted for studies reporting on mucocutaneous disease manifestations as primary outcome, followed by those investigating rheumatological outcomes, then ocular outcomes. There are currently no high quality controlled trials of therapy for neurological consequences of BD (Figure 4).

**Figure 5:**
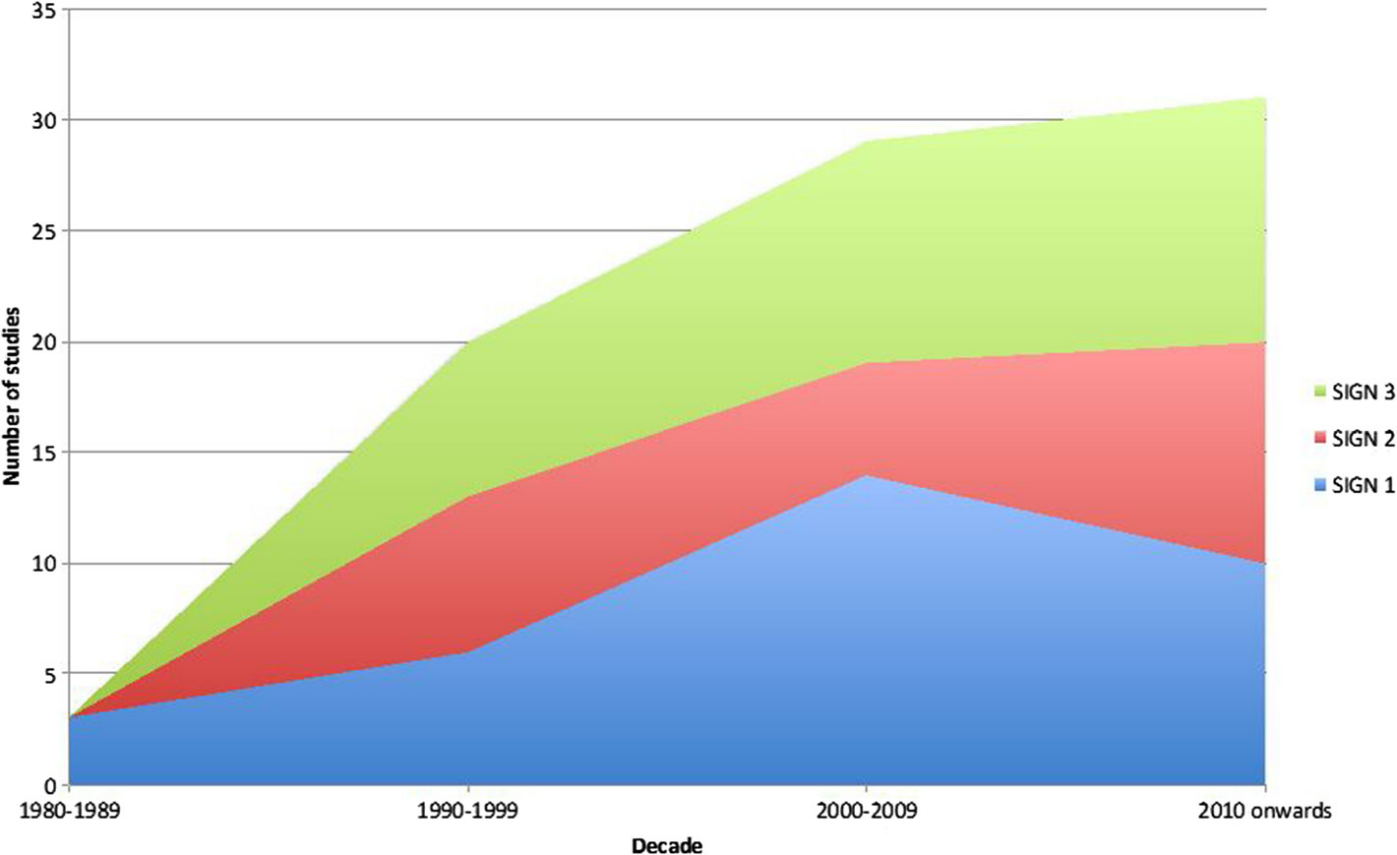
SIGN score by decade 1988-2013.

There has been a relative decline in the number of SIGN-1 graded studies being published since 2010, compared to a relative increase in lower-quality grade 2 and 3 studies (Figure 5).

## Discussion

Evidence-based practice demands continuous reappraisal of peer-reviewed literature, and clinicians must be prepared to update treatment guidelines as new therapies are validated. To achieve this, it is useful to assess the scope of published data, both to review those disease manifestations and treatment modalities that have been thoroughly investigated, and identify any which may have been neglected. Through review of current trends in published research we can predict how the situation is likely to evolve over time and identify areas of unmet need for future investigation.

This report represents the first such assessment of trends over time in published literature for the management of BD. We have reviewed the available data with respect to disease manifestations investigated, therapeutic interventions tested and have made an assessment of the quality of this evidence.

We conclude that whilst there is a wealth of published literature available to guide evidence-based practice in BD, there are still worrying gaps in the evidence:

In our analysis, mucocutaneous and ocular manifestations were the most commonly reported primary outcomes, followed by rheumatological disease, with very few studies reporting on neurological or cardiovascular symptoms as primary outcome. These proportions reflect the prevalence of each manifestation in the patient population; in one review mucocutaneous disease was reported in almost all patients, ocular involvement in up to 69%, joint involvement in up to 59%, vascular involvement in up to 38% and central nervous system involvement in up to 20% of patients with BD [6]. It could thus be reasoned that “supply” of literature is largely matched to “demand”.

Review of publication trends over time suggests that whilst the number of studies reporting ocular manifestations as primary outcome are increasing, there have been no new publications using mucocutaneous manifestations as primary outcome since 2009, and none using rheumatological manifestations as primary outcome since 1998. Extrapolating these trends forwards, this suggests a “plateau” in new data generation for certain aspects of disease, with little new data likely to appear in the foreseeable future.

Furthermore, whilst mucocutaneous disease is indeed the most common manifestation of BD, it is the cardiovascular and neurological disease which has the potential to be most serious [5]. Currently, the ability to make evidence-based treatment decisions for neurological and cardiovascular disease manifestations is severely limited by a lack of data, with only four studies employing cardiovascular disease as primary outcome and two neurological disease.

We found no literature to guide interventions in the management of gastro-intestinal disease. This has previously been documented [70] and we find no evidence of changing practice in this area.

The current trend in the types of therapeutic agent assessed reveals a bias towards newer, biologic therapies, with a relative over-representation of studies testing biologics in ocular disease. Again, this reflects the relative frequency and severity of ocular manifestations in BD.

There is a paucity of studies at the highest levels of scientific evidence with over-representation of SIGN-2 and SIGN-3 graded papers. This is particularly noticeable in those studies reporting primary outcomes of ocular disease, which whilst showing the greatest increase in frequency, display one of the lowest incidences of SIGN-1 graded studies. There is currently no SIGN-1 graded literature for management of neurological disease.

Again, it is perhaps most worrying that we observe a relative decrease in the overall frequency of SIGN-1 graded papers over recent years, whilst the frequency of lower quality papers is increasing. Our exclusion criteria eliminated all SIGN-4 graded papers from review.

Given the deficit of published data to guide management of the less common manifestations of BD and the difficulty in performing large-scale randomized controlled trials in rare diseases, clinicians may wish to consider an alternative approach to generation of treatment guidelines. Kobayashi *et al*. employed a modified Delphi approach in developing guidelines for the diagnosis and management of intestinal BD [71]; this approach involves development of consensus-based guidelines through expert review of a selection of clinical statements extracted from existing literature. It could be argued that rather than being limited to the conventional hierarchy of evidence as described by SIGN, this approach allows a degree of objective assessment of lower-quality studies which would traditionally be excluded from published systematic reviews.

For rare diseases, consensus-based guidelines may necessitate an international collaborative approach, which can be facilitated through centres of excellence [72]. In addition to “physical” centres of excellence in a hospital setting, there is a growing recognition of the value of virtual centres of excellence such as the ORPHANET information portal [73].

We have made no comment on treatment efficacy or side effects in our review. Hatemi *et al*. have previously published detailed guidelines for the management of BD, both in the form of evidence-based recommendations by The European League Against Rheumatism (EULAR) [74], and an accompanying systematic review of published literature [70]. An independent management algorithm was proposed in a wider review article by Alpsoy in 2012 [75], and a Cochrane review of pharmacotherapy in BD was published in 2009 [76]. These publications currently constitute the “gold standard” for clinicians wishing to review treatment outcomes in existing literature, and provide a strong foundation on which to base treatment decisions.

Clinicians should however be aware of the limitations of these existing reviews: The EULAR review assessed published literature only up to December 2006 and Alpsoy up to 2010, whilst the Cochrane review (despite being published later) included RCTs up to January 1998. Furthermore, whilst the Alpsoy review is a useful resource, it makes no comment on the quality of referenced studies, and relies on a mixture of primary data and existing review articles. According to our inclusion criteria, we have identified a further 21 studies which were published since the EULAR recommendations were finalized, and a further 16 RCTs since the termination of the Cochrane review in 1998. Our review would suggest an update of each of these existing sources is indicated.

We also note that these existing reviews assess only pharmacological treatment options; high-quality evidence is lacking in other modalities, however we found evidence of ten studies reporting either primary or secondary study outcomes of non-pharmacological therapies. Whilst we acknowledge that surgery and other invasive interventions should be reserved as a last resort, we would urge clinicians to remember the impact of diet and exercise on general health and well being of patients with BD, and consider these areas which would benefit from increased research activity.

This review is subject to a number of limitations: As discussed above, we have made no comment on the detail of study outcomes, and have only categorized them in terms of organ system investigated. We would recommend that clinicians refer to the aforementioned publications by Hatemi *et al*. for a comprehensive discussion of treatment efficacy [70,74], but remain aware of the need for frequent updates of published guidelines and be prepared to consult original sources for themselves.

We may also have excluded a number of “useful” publications based on their small sample size; Behçet’s Disease remains relatively rare in many populations [5] and clinicians may struggle to generate large sample sizes for clinical studies. They may however generate interesting data on unusual treatment modalities in small groups, and we would encourage clinicians to assess these studies for themselves.

## Conclusions

We conclude that evidence-based practice in BD is currently an ideal and not a feasible reality. To date, there have been numerous studies on treatment for ocular and mucocutaneous manifestations, however the availability of evidence from large scale, randomized controlled trials remains limited and we hope investigation continues in these areas. There is an alarming shortage of research investigating all other disease manifestations, and this is an important area of unmet need that should be addressed through future work.

In addition, whilst current research is biased towards newer biologic therapies, there is significant scope for reappraisal of older agents that may prove to be beneficial in certain situations following rigorous scientific review.

## Abbreviations

BD: Behçet’s disease; EBM: Evidence based medicine; EULAR: European league against rheumatism; ISG: International study group; SIGN: Scottish intercollegiate guideline network.

## Competing interests

The authors declare that they have no competing interests.

## Authors' contributions

BM, RM, HK, NJ and MM performed the initial scoping study to identify papers for inclusion in this analysis and generated preliminary results on which this manuscript is based. RB reviewed this initial scoping study, finalized the list of papers for inclusion, completed the final analyses and produced the manuscript. AD and PM conceived of the study, and participated in its design and coordination and helped to draft the manuscript. All authors read and approved the final manuscript.
